# Phosphinegold(I) Thiolates – Pharmacological Use and Potential

**DOI:** 10.1155/S1565363303000050

**Published:** 2003

**Authors:** Edward R. T. Tiekink

**Affiliations:** Department of Chemistry National University of Singapore 117543 Singapore

## Abstract

A brief overview of the use of gold compounds in medicine, namely in the treatment of rheumatoid arthritis is presented, including that of the orally-administered triethylphosphinegold(I) tetraacetylatedthioglucose species, auranofin. A summary of an evaluation for anti-arthritic activity of novel phosphinegold(I) thiolate analogues containing biologically active thiols is given. This shows that compounds with greater efficacy and reduced toxic side-effects, at least in the in vivo model studied, can be developed. More recent investigations on this class of compound have focussed on their putative anti-tumour activity. Significant cytotoxicity and apparent cellular specificity have been discovered for certain phosphinegold(I) thiolates carrying water-solubilising groups. All indications are there that the continued exploration of the medicinal properties of phosphinegold(I) thiolates offers very real opportunities in metallotherapeutics.


### Phosphinegold(I) Thiolates – Pharmacological Use and Potential

Edward R.T. Tiekink

*Department of Chemistry, National University of Singapore, Singapore 117543*
*Tel.: 65 6874 2848; Fax.: 65 6779 1691; e-mail: chmtert@nus.edu.sg*

(Received: October 30, 2002; Accepted: November 21, 2002)

### ABSTRACT

A brief overview of the use of gold compounds in medicine, namely in the treatment of rheumatoid arthritis is presented, including that of the orally-administered triethylphosphinegold(I) tetraacetylatedthioglucose species, auranofin. A summary of an evaluation for anti-arthritic activity of novel phosphinegold(I) thiolate analogues containing biologically active thiols is given. This shows that compounds with greater efficacy and reduced toxic side-effects, at least in the *in vivo* model studied, can be developed. More recent investigations on this class of compound have focussed on their putative anti-tumour activity. Significant cytotoxicity and apparent cellular specificity have been discovered for certain phosphinegold(I) thiolates carrying water-solubilising groups. All indications are there that the continued exploration of the medicinal properties of phosphinegold(I) thiolates offers very real opportunities in metallotherapeutics.

**Keywords:** Gold, thiolate, phosphine, rheumatoid arthritis, anti-tumour activity, chrysotherapy, metal-based drugs

### INTRODUCTION

The use of metal compounds in medicine is now well established. This is no better evidenced than by the appearance of comprehensive reviews of the subject, *e.g.* /1, 2/. Thus, metal compounds containing bismuth, gold, and platinum are well known as therapeutic agents against peptic ulcers, rheumatoid arthritis and various forms of cancer, respectively. While this list is by no means exhaustive, it is also important to realise that many metal compounds containing different elements are undergoing preliminary and clinical trials, suggesting that new metal-based drugs are imminent. Further, metal compounds find wide use as imaging agents /1, 2/. Amongst the oldest metals used in medicine, for real or putative treatments, is gold and this element forms the focus of the present review.

The mystique associated with the noblest of all elements, gold, has ensured it has played a role in medicine for thousands of years. Indeed, gold has been recorded as being used to treat a variety of ailments by Chinese and Arabic physicians since 2500 BC. In more modern times, a scientific basis for employing gold compounds was established by Robert Koch as it was his work that established the bacteriostatic properties of gold cyanide preparations; for a fascinating account of the historical development of gold in medicine, the reader is referred to ref. /3/. Since then, gold compounds have demonstrated the ability to ameliorate the symptoms associated with rheumatoid arthritis e.g. /4, 5/; see below. Given the widespread use of metal-based drugs, it is perhaps not surprising that gold compounds have been investigated for other medicinal applications. Arguably, most attention in this regard has been devoted to discovery of anti-tumour activity of both gold(I) and gold(III) compounds e.g. /6/. Over and above this, gold compounds have been investigated for the treatment of parasitic diseases such as malaria e.g. /7/ and Chagas disease e.g. /8/. A number of gold compounds have exhibited some anti-HIV activity e.g. /9/ and older work has suggested that some gold compounds used in the treatment of rheumatoid arthritis may be utilised in the treatment of bronchial asthma e.g. /10/. Given the use and potential use of gold compounds in medicine, it is perhaps surprising that the mechanism(s) of action of gold drugs is not very well understood and is, in fact, subject to contention. A recent review covers many aspects of the biochemistry of gold and its compounds, and is highly recommended reading /11/. Despite this lack of understanding of the precise mechanism of action of gold drugs, it is the known anti-arthritic activity and putative anti-tumour activity that motivates our studies in this area and forms the emphasis of this review.

Our research in this field was initiated by the desire to develop new phosphinegold(I) thiolates that could supplant the currently used gold-based anti-arthritic agents. Based on encouraging early results, in particular the relatively high mammalian tolerance of our compounds, and prompted by commercial advice, the focus of our research turned to the examination of the anti-tumour potential of the newly synthesised compounds. In this review, a brief summary of the attempted development of new anti-arthritic agents will be presented that is followed by a summary of our research into the anti-tumour activity of phosphinegold(I) thiolates.

### PHOSPHINEGOLD(I) THIOLATES AS ANTI-ARTHRITIC AGENTS

*Chrysotherapy* is the term given to describe the use of gold compounds in medicine. Therefore, this term refers to the treatment of rheumatoid arthritis as, to date, gold compounds are not used in any other therapeutic applications. There are a relatively large number of gold compounds used in this context and they can be conveniently divided into two classes, reflecting their mode of administration. The first therapeutic gold drugs were the polymeric gold thiolates. Chemical structures for these are shown in Fig. 1. The compounds are water-soluble, being charged as in sanocrysin (**1**, aurothiosulphate), allocrysin (**2**, aurothiopropanol sulphonate), and arguably the most widely-used gold drug, myocrisin (**4**, aurothiomalate) or by carrying water-solubilising functionality as in solganol (**3**, aurothioglucose), so that their mode of administration is normally *via* injection, usually on a weekly basis. A more recent addition to *chrysotherapy* is the decidedly more lipophilic compound, auranofin (5, (1-thio-β-D-glucopyranose-2,3,4,6-tetraacetato-*S*)-(triethylphosphine)gold(I)), as shown in Fig. 2, which is an example of a phosphinegold(I) thiolate. Owing to its lipophilic nature, auranofin is administered orally on a daily basis. In common with other forms of metallotherapy, the administration of gold drugs inevitably results in a number of deleterious side-effects such as nausea, dermatitis, nephritis, *etc.* Further, not all patients respond to *chrysotherapy*. With the above in mind, there is a definite need for more effective gold compounds for the treatment of rheumatoid arthritis and compounds with reduced toxic side-effects. Our contributions in this area were motivated by exploiting the relatively little knowledge that was known of the mechanism of action of gold drugs.

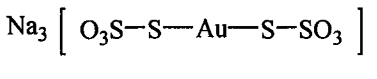

**1**

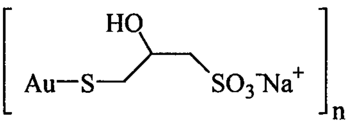

**2**

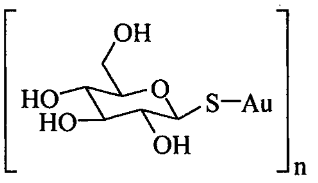

**3**

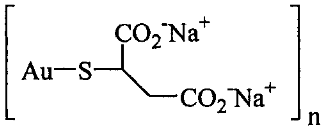

**4**

**Fig. 1:** Chemical structures of gold thiolates used in the treatment of rheumatoid arthritis: (**1**) sanocrysin (aurothiosulphate), (**2**) allocrysin (aurothiopropanol sulphonate), (**3**) solganol (aurothioglucose), and (**4**) myocrisin (aurothiomalate).

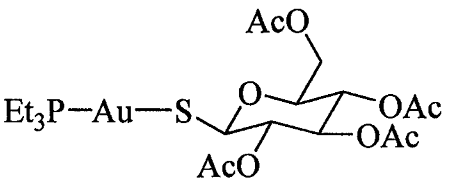

**5**

**Fig. 2:** Chemical structure of an orally administered phosphinegold(I) thiolate used in the treatment of rheumatoid arthritis: (**5**) auranofin.

It has been established that gold drugs are in fact pro-drugs, as the original gold compounds are rapidly metabolised *in vivo* in order to generate the pharmacologically active species /11/. For example, auranofin (5) is known to react with the ubiquitous blood protein, albumin, at the cysteine-34 site, with displacement of the tetraacetylatedthioglucose moiety by the protein. Subsequent reaction of the gold compound with other thiols such as albumin or glutathione results in liberation of triethylphosphine, which is rapidly oxidised to its oxide with concomitant reduction of the disulphide link in albumin. It is this way, it is clear that the gold compounds serve as a carrier to deliver gold so as to form a gold/protein complex. Given this information, it seemed obvious to couple biologically active thiols, *i.e.* thiols with known anti-arthritic profiles, with phosphinegold(I) entities so that upon administration, both the "gold" and liberated thiol could provide therapeutic advantage. With this in mind, thiols such as 6-mercaptopurine (**6a**) and 6-thioguanine (**6b**), molecules with known anti-arthritic activity /12/, were complexed to phosphinegold(I) to form species represented as e.g. 7; see Fig. 3 for chemical structures. Given that the protocols associated with anti-arthritic screening are not widely known, it is appropriate that a brief outline be presented here.

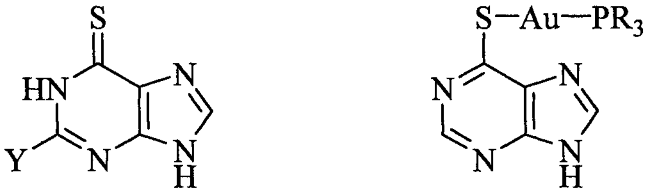

**6a**: Y = H
**6b**: Y = $NH_2$

7; R = alkyl, aryl

**Fig. 3:** Chemical structures (**6a**) mercaptopurine (6-MPH), (**6b**) 6-thioguanine (6-TGH), and (7) $R_3PAu$(6-MP).

*In vivo* screening for anti-arthritic activity was conducted in an animal model developed specifically for this purpose /13, 14/. The dark Agouti rat model is a gold-sensitive rat strain that most closely matches human response to gold drugs. Typically, rats have arthritic symptoms induced by the application of arthritogenic Freund's adjuvant into the tail-base. A sub-set of these animals has concomitant administration of the trial gold compound. Differences in physical appearance are then assessed so to as to determine potency and toxicity of the gold compounds.

An example of a biological trial is shown in Fig. 4 which shows the lower parts of three dark Agouti rats. Here, the animal on the left is the control, having neither induced arthritis nor administration of gold compound. The animal on the right has had arthritis induced as can be readily judged by paw and tail swelling. The animal in the middle has had arthritis induced by Freund's adjuvant arthritis and at the same time has been administered with a gold compound so that reduced paw and tail swelling is evident. The determination of the efficacy and toxicity is based on qualitative assessments.

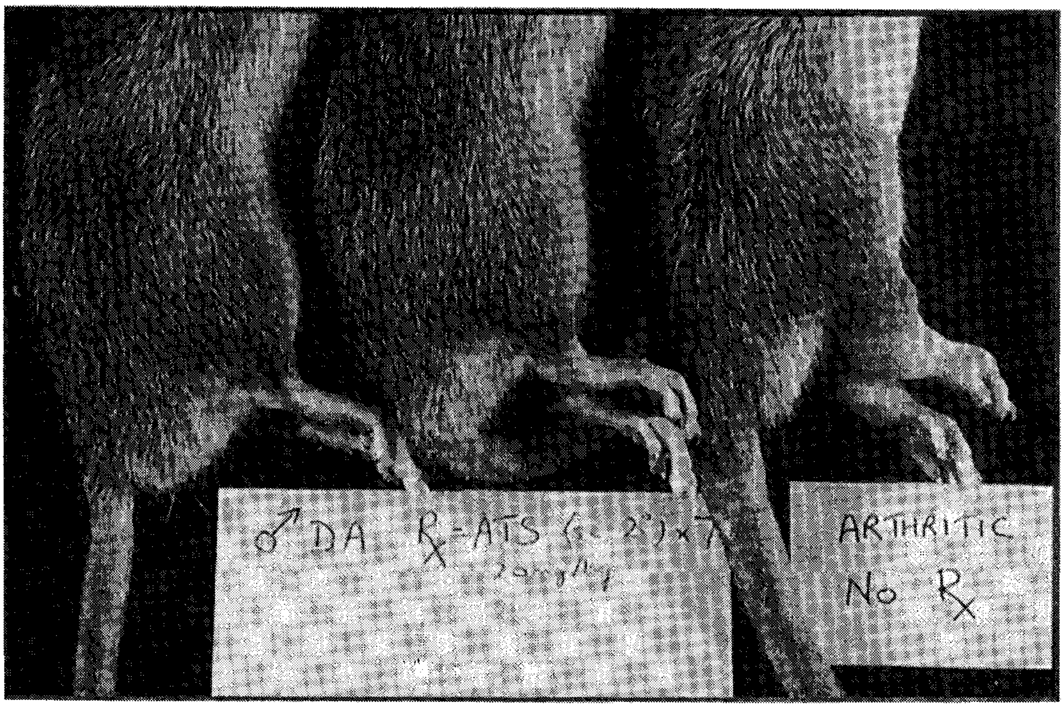

**Fig. 4:** Lower regions of three dark Agouti rats used in the screening for anti-arthritic activity. The animal on the left is a healthy specimen, the animal in the middle is an arthritic animal with administered gold drug and that on the right is an arthritic animal; after /16/..

Potency is scored on a rating of '+' to '4+'. A score of 3+ corresponded to treatments that had completely suppressed polyarthritis on day 18. The highest rating of 4+ equated to the situation where no arthritis had developed by day 28. Toxicity is rated from '-' to '2+', with the latter value being regarded as unacceptable. Toxicity was judged by considering weight loss, diarrhoea and albuminuria in both treated and untreated animals. Table 1 summarises potency and toxicity ratings for a series of phosphinegold(I) mercaptopurinates (Fig. 3), for related thiolates (see Fig. 5 for structures of thiols), and for the gold drugs aurothiomalate (**4**) and auranofin (**5**) /15, 16/.

From the data presented in Table 1 a number of conclusions can be made. First and foremost, several of the compounds exhibit anti-arthritic potency to a far greater extent than auranofin (**5**), recalling that ratings of 4+ correspond to no signs of the disease at day 28, after which animal ethics regulations dictated that the animal must be sacrificed. Further, many of the compounds had toxicity ratings significantly lower than that for **5**. Comparing the new results with those obtained for aurothiomalate, for which toxicity is not as great as for **5**, many of the compounds listed had higher potency ratings.

It was mentioned above that 6-mercaptopurine (**6a**) and 6-thioguanine (**6b**) have exhibited anti-arthritic activity in their own right. So it is pertinent to recognise that complexation to phosphinegold(I) resulted in more potent compounds thereby demonstrating the importance of gold for therapeutic applications. The most active compounds were indeed those containing the deprotonated forms of **6a** and **6b**. Other derivatives

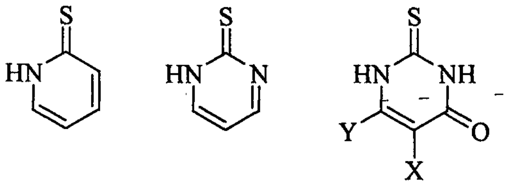

**8** **9** **10a**: X = Y = H

**10b**: X = $CO_2H$, Y = H

**10c**: X = H, Y = Me

**10d**: X = Y, Y = nPr

**Fig. 5:** Chemical structures of the thiols relevant to the data presented in Table 1: (**8**) 2-mercaptopyridine (2-pySH), (**9**) 2-mercaptopyrimidine (2-pymS), (**10a**) 2-thiouracil (2-TUH), (**10b**) 5-carboxy-2-thiouracil (5-$CO_2H$-2-TUH), (**10c**) 6-methyl-2-thiouracil (6-Me-2-TUH) and (**10d**) 6-n-propyl-2-thiouracil (6-nPr-2-TUH).

**Table 1.**

Potential therapeutic activity in dark Agouti rats - a screen for anti-arthritic activity.[A]

| Compound | Toxicity | Anti-arthritic potency | Ref. |
|---|---|---|---|
| $Et_3PAu$(6-MP) | - | 4+ | /15/ |
| $Ph_3PAu$(6-MP) | + | 3+ | /15/ |
| $Cy_3PAu$(6-MP) | + | 4+ | /15/ |
| $Et_3PAu$(6-TG) | + | 2+ | /15/ |
| $Ph_3PAu$(6-TG) | 2+ | 4+ | /15/ |
| $Cy_3PAu$(6-TG) | + | 3+ | /15/ |
| $Ph_3PAu$(2-pyS) | + | 0 | /16/ |
| $Ph_3PAu$(2-pymS) | 2+ | 4+ | /16/ |
| $Cy_3PAu$(2-pymS) | 2+ | 3+ | /16/ |
| $Cy_3PAu$(2-TU) | 2+ | + | /16/ |
| $Ph_3PAu$(5-$CO_2H$-2-TU) | 2+ | 4+ | /16/ |
| $Cy_3PAu$(5-$CO_2H$-2-TU) | 0 | 3+ | /16/ |
| $Ph_3PAu$(6-Me-2-TU) | + | 3+ | /16/ |
| $Ph_3PAu$(6-nPr-2-TU) | 2+ | 4+ | /16/ |
| $Cy_3PAu$(6-nPr-2-TU) | + | 2+ | /16/ |
| Aurothiomalate (**4**) | - | 2+ | /16/ |
| Auranofin (**5**) | 2+ | 2+ | /16/ |

*A* The structures and abbreviations of the thiols included in this Table are given in Figs 3 and 5.

containing thiolates derived from 2-thiouracil, 2-mercaptopyridine, and other thiols (see Fig. 6 for chemical structures) were not as potent or were more toxic /16, 17/ and so their study was not pursued.

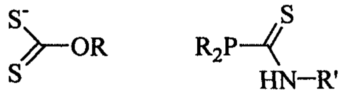

11 12

R, R' = alkyl, aryl

**Fig. 6:** Chemical structures of sulphur-containing ligands for which their gold compounds have been screened for anti-arthritic activity: (**11**) alkyl (aryl) xanthate and (**12**) phosphinothioformamide.

Of all the compounds screened, the most potent and least toxic was $Et_3PAu$(6-MP), having potency and toxicity ratings of 4+ and -, respectively. This compound has the same phosphine ligand as found in auranofin, *i.e.* triethylphosphine. However, no definitive structure/activity relationship may be established as for other series of compounds, different phosphine ligands were incorporated in the most active compounds. These observations suggest that it is a subtle combination of both thiolate and phosphine ligands that gives rise to the more active compounds. As an example of this point, a series triorganophosphinegold(I) xanthates (see Fig. 6 for the structure of the xanthate anion), *i.e.* $R_3PAu(S_2COMe)$, were evaluated /16/. For the R = Ph and Cy compounds, the potency was rated as + and 3+, respectively. By contrast, the R = Et compound was lethal.

From the above discussion and data presented in Table 1, it is clear that some of the compounds possess potency and toxicity profiles that, at a minimum, rival those exhibited by the commercially available drugs. A particularly exciting feature of the above studies was the observation that relatively high doses of 'gold' could be tolerated by the dark Agouti rats, *i.e.* up to 10 mg/kg every two days for up to 9 doses suggesting a high-level of tolerance. Further studies in putative biological activity have focussed on anti-tumour activity for essentially the same reasons as given for the study of these compounds in the first place. Thus, not only do the thiols mercaptopurine (**6a**) and 6-thioguanine (**6b**) exhibit immunosuppressant activity, they display antineoplastic activity /12/ and so it was thought worthwhile to investigate the cytotoxicity/anti-tumour activity of phosphinegold(I) thiolates.

### CYTOTOXICITY AND ANTI-TUMOUR ACTIVITY OF PHOSPHINEGOLD(I) THIOLATES

There are in fact several good reasons for exploring the anti-tumour potential of phosphinegold(I) thiolates. One obvious reason is as given above, *i.e.* that the complexation of biologically active thiols to gold will alter the metabolic pathways of the thiols and/or provide a slow-release mechanism of the thiol, either of which might lead to different profiles of anti-tumour activity. Thus, one might regard the gold as a 'platform' or carrier for the effective delivery of a thiol for therapeutic effect. Another justification for investigating gold compounds in this context is found from the medical literature. Long-term studies established a possible connection between *chrysotherapy* and anti-tumour activity. In this way, evidence was presented that suggested that patients undergoing *chrysotherapy* were less likely to suffer from cancer disease /18/. This finding inevitably led to the investigation of anti-tumour activity of gold compounds used in the treatment of rheumatoid arthritis. Of these, auranofin (**5**) proved to be the most interesting and was subjected to further evaluation.

Auranofin (**5**) was proven to possess significant *in vitro* activity /19-21/ and this was translated into animal models but only for P388 leukaemia inoculated in mice /22, 23/. Not surprisingly, many auranofin derivatives were subsequently investigated for cytotoxicity and anti-tumour activity /24/. This early work unveiled a number of interesting conclusions that still direct on-going studies in this field. Arguably, the key result of this study was the establishment of the following principle: compounds that were active *in vitro* were not necessarily active *in vivo* but compounds that were not active *in vitro* were unlikely to be active *in vivo*. Hence, at least for the gold compounds investigated, a protocol was established that allowed the rapid and economical screening of gold compounds for anti-tumour potential. In terms of a possible structure/activity relationship, several clues were uncovered /24/.

It was shown that phosphinegold(I) thiolates were more active than gold thiolates, indicating the importance of the phosphine ligand for activity. In the same way, phosphinegold(I) thiolates had more promising cytotoxicity profiles than their chloride analogues, showing that, potentially, the thiolate ligand also plays a role in cytotoxicity/anti-tumour activity /24/. In summary, and important in the context of the present overview, this study indicated the potential of phosphinegold(I) thiolates as anti-tumour agents. Despite this, the immediate focus of subsequent studies was upon the bis-chelated, tetrahedrally coordinated gold(I) compound, $[Au(dppe)_2]^+$ (**13**, dppe is bis(diphenylphosphino)ethane, see Fig. 7 for chemical structure), an often observed rearrangement product from precursors such as XAu(μ-dppe)AuX, and analogues, as these showed promising activity e.g. /25/. Clinical trials for $[Au(dppe)_2]^+$ were precluded owing to acute toxicity in organs such as the heart, liver and lungs /26/. Nevertheless, studies continue with analogues of $[Au(dppe)_2]^+$, with the purpose of targeting mitochondrial DNA /27/. Biological evaluation of phosphinegold(I) thiolate species continues owing to the promising activity exhibited by the phosphinegold(I) thiolates as reported above.

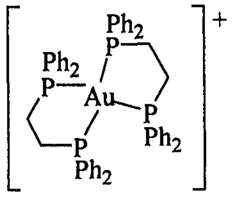

**13**

**Fig. 7:** Chemical structure of the bis-chelated, tetrahedrally coordinated gold(I) compound, $[Au(dppe)_2]^+$ (**13**).

Besides the aforementioned studies, the first work published describing investigations of the potential anti-tumour activity of phosphinegold(I) thiolates was published in the early 1990's /28-30/. The triphenylphosphinegold(I) compound of the anion derived from thiotheophylline (14, 8-mercapto-1,3-dimethyl-3,7-dihydro-purine-2,6-dione; see Fig. 8) has demonstrated potency against L1210 leukaemia, B16F10 melanoma, and in Friend leukaemia cells in culture, and shows greater activity than the uncomplexed ligand /28/, again demonstrating the importance of gold for efficacy. A possible indication to the mode of action of phosphinegold(I) thiolates was indicated by the observation that the compound was readily incorporated into the cytoplasm and in the nuclear fraction so as to induce single-stranded breaks in DNA /29, 30/. Our work in this field has been motivated by the detection of cytotoxicity/anti-tumour activity of phosphinegold(I) thiolates described above. A particular focus has been to couple biologically-active thiols with phosphinegold(I) entities in the hope that upon administration of the 'pro-drug', both the phosphinegold(I) entity and thiol would provide therapeutic benefit.

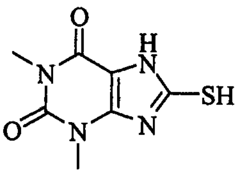

**14**

**Fig. 8:** Chemical structure of thiotheophylline (**14**).

The first cytotoxicity screens in this aspect of our study /31, 32/ were conducted on compounds related to those described above for the anti-arthritic assays. Selected data are collated in Table 2; see Figs 3 and 5 for chemical structures. From this data, it is evident that the presence of the phosphinegold(I) entity enhances the overall cytotoxicity of the thionucleobases studied, some of which display activity against leukaemia /12/. In the case of the triphenylphosphinegold(I) species, the importance of having thiolate present is also apparent as triphenylphosphinegold(I) chloride is less cytotoxic, at least in the cell lines examined. All of the phosphinegold(I) compounds listed have cytotoxicities greater than the standard compound, cisplatin, in a range of cancer cell lines: mouse leukaemia, human squamous cell carcinoma and human ovarian carcinoma. This high-level of cytotoxicity is particularly noticeable against the cisplatin-resistant mouse leukaemia cell line. These results were of sufficient interest to warrant further investigation.

*In vivo* studies conducted on a cisplatin-sensitive PC6 plasmacytoma inoculated in mice revealed that cisplatin cured all mice. By contrast, the 6-MPH (at 50 mg/kg) and $Ph_3PAuCl$ (at 25 mg/kg) compounds did not reduce the size of the tumour. More effective was $Ph_3PAu$(6-MP) (at 25 mg/kg) which led to a 60% reduction in tumour size /32/. The cytotoxicity profiles of four compounds were also evaluated against the National Cancer Institute's (U.S.A.) panel of 60 cell lines /33/. Thus, an order of cytotoxicity was established for the $R_3PAu$(6-MP) series such that R = Cy > Ph > Et. The other compound examined was $Et_3PAu$(6-TG) and amongst all four compounds, $Cy_3PAu$(6-MP) was the most cytotoxic. Finally, sub-panel selectivity

**Table 2**

Cytotoxicity ($IC_{50}$, μM) of phosphinegold(I) thiolates and thionucleobases.[A, B]

| Compound | L1210 | L1210/DDP | FaDu | SKOV-3 | Ref. |
|---|---|---|---|---|---|
| $Et_3PAu$(2-TU) | 0.086 | | | | /31/ |
| $Ph_3PAu$(2-TU) | 0.131 | | | | /31/ |
| $Cy_3PAu$(2-TU) | 0.115 | | | | /31/ |
| $Et_3PAu$(6-MP) | 0.094 | | | | /31/ |
| $Ph_3PAu$(6-MP) | 0.083 | 0.05 | 0.25 | 0.36 | /31, 32/ |
| $Cy_3PAu$(6-MP) | 0.079 | | | | /31/ |
| $Et_3PAu$(6-TG) | 0.041 | | | | /31/ |
| $Ph_3PAu$(6-TG) | 0.052 | n/a | 0.39 | 0.63 | /31, 32/ |
| $Cy_3PAu$(6-TG) | 0.084 | | | | |
| 2-TUH | > 100 | | | | /31/ |
| 6-MPH | 0.310 | 0.33 | > 5 | 19.5 | /31, 32/ |
| 6-TGH | 0.30 | n/a | > 2 | 9.1 | /31, 32/ |
| $Ph_3PAuCl$ | 0.096 | 0.14 | n/a | 0.13 | /32/ |
| cisplatin | 0.6 | 6.7 | 6.1 | 3.1 | /31, 32/ |

[A] The structures and abbreviations of the thiols included in this Table are given in Figs 3 and 5.

[B] Abbreviations for cancer cell lines: L1210, mouse leukaemia; L1210/DDP: cisplatin-resistant mouse leukaemia; FaDu, human squamous cell carcinoma; SKOV-3, human ovarian carcinoma; and n/a: not screened.

against the leukaemia cell lines was found for both $Cy_3PAu$(6-MP) and $Et_3PAu$(6-TG) /33/. Further studies have focussed upon increasing the aqueous solubility of the phosphinegold(I) thiolates. A difficulty that was identified for the aforementioned 6-MP and 6-TG compounds was their insolubility in aqueous medium /34/. Hence, compounds with water-solubilising functionality have been developed and these, too, have been shown to exhibit exciting cytotoxicity profiles.

Subsequent work involved a panel of seven human cell lines as indicated in Tables 3 and 4 /35-37/. In Table 3, results are listed for $Ph_3PAu$(6-MP), to allow for reference to previous work, two new compounds, *i.e.* $R_3PAu(SC(=O)Ph)$ for R = Ph and Cy, and a series of established cytotoxic agents, namely cisplatin, doxorubicin, 5-fluorouracil, methotrexate, etoposide and taxol /35/; structures for the non-metal containing compounds are shown in Fig. 9. The gold compounds display moderate to high toxicity, especially compared to cisplatin. While there is an obvious attraction to develop compounds with greater cytotoxicity profiles than cisplatin, it is also imperative that the compounds are more active than the established 'organic' drugs. Against this criterion, the investigated phosphinegold(I) thiolates were not particularly promising. Another key objective of drug design is to generate compounds with specificity in activity, *i.e.* compounds that target specific cell lines. The above aims have been realised, at least based on *in vitro* studies, for the next series of compounds to be discussed.

**Table 3**

Cytotoxicity ($ID_{50}$, ng/ml) data for $Ph_3PAu(SC(=O)Ph)$, $Cy_3PAu(SC(=O)Ph)$, $Ph_3PAu(6\text{-}MP)$ and established cytotoxic agents /35/ against a panel of seven human cancer cell lines.[A, B]

| Compound | A498 | EVSA-T | H226 | IGROV | M19 | MCF-7 | WIDR |
|---|---|---|---|---|---|---|---|
| $Ph_3PAu(SC(=O)Ph)$ | 954 | 826 | 1124 | 324 | 1031 | 1010 | 942 |
| $Cy_3PAu(SC(=O)Ph)$ | 4358 | 3319 | 6293 | 2916 | 2805 | 2805 | 5135 |
| $Ph_3PAu(6\text{-}MP)$ | 1236 | 456 | 738 | 347 | 915 | 943 | 1200 |
| cisplatin | 2253 | 422 | 3269 | 169 | 558 | 699 | 967 |
| 5-FU | 143 | 475 | 340 | 297 | 442 | 750 | 225 |
| TAX | < 3.2 | < 3.2 | < 3.2 | < 3.2 | < 3.2 | < 3.2 | < 3.2 |
| DOX | 90 | 8 | 199 | 60 | 16 | 10 | 11 |
| MTX | 37 | 5 | 2287 | 7 | 23 | 18 | < 3.2 |
| ETO | 1314 | 317 | 3934 | 580 | 505 | 2594 | 150 |

[A] Abbreviations and structures of the established cytotoxic agents are given in Fig. 9.

[B] Abbreviations for the human cancer cell lines: A498, renal cancer; EVSA-T, estrogen receptor (ER)-/progesterone receptor (PgR)-; H226, non-small cell lung cancer; IGROV, ovarian cancer; M19, melanoma; MCF-7, estrogen receptor (ER)+/progesterone receptor (PgR)+; and WIDR, colon cancer.

**Table 4**

Cytotoxicity ($ID_{50}$, ng/ml) data for $R_3PAu(n\text{-}SC_6H_4CO_2H)$, R = Et, Cy & Ph; n = 2, 3 & 4, and established cytotoxic agents /36-37/ against a panel of seven human cancer cell lines.[A, B]

| Compound | A498 | EVSA-T | H226 | IGROV | M19 | MCF-7 | WIDR |
|---|---|---|---|---|---|---|---|
| $Et_3PAu(2\text{-}SC_6H_4CO_2H)$ | 65 | 110 | 69 | 43 | 118 | 405 | 984 |
| $Cy_3PAu(2\text{-}SC_6H_4CO_2H)$ | 95 | 225 | 97 | 182 | 292 | 411 | 772 |
| $Ph_3PAu(2\text{-}SC_6H_4CO_2H)$ | 151 | 356 | 173 | 117 | 298 | 791 | 982 |
| $Cy_3PAu(3\text{-}SC_6H_4CO_2H)$ | 697 | 1046 | 848 | 283 | 403 | 806 | 949 |
| $Ph_3PAu(3\text{-}SC_6H_4CO_2H)$ | 1274 | 1287 | 960 | 280 | 797 | 1065 | 957 |
| $Et_3PAu(4\text{-}SC_6H_4CO_2H)$ | 484 | 1197 | 864 | 67 | 375 | 1145 | 1238 |
| $Cy_3PAu(4\text{-}SC_6H_4CO_2H)$ | 743 | 1353 | 923 | 338 | 592 | 973 | 976 |
| $Ph_3PAu(4\text{-}SC_6H_4CO_2H)$ | 348 | 940 | 586 | 148 | 322 | 858 | 553 |
| cisplatin | 2253 | 967 | 3269 | 558 | 169 | 422 | 699 |
| 5-FU | 143 | 225 | 340 | 442 | 297 | 475 | 750 |
| DOX | 90 | 11 | 199 | 16 | 60 | 8 | 10 |
| MTX | 37 | < 3 | 2287 | 23 | 7 | 5 | 18 |
| ETO | 1314 | 150 | 3934 | 505 | 580 | 317 | 2594 |

[A] Abbreviations and structures of the established cytotoxic agents are given in Fig. 9.

[B] Abbreviations for the human cancer cell lines are given in the caption to Table 3.

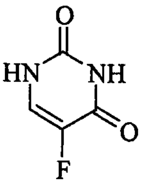

**15**

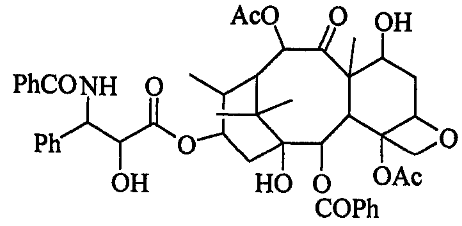

**16**

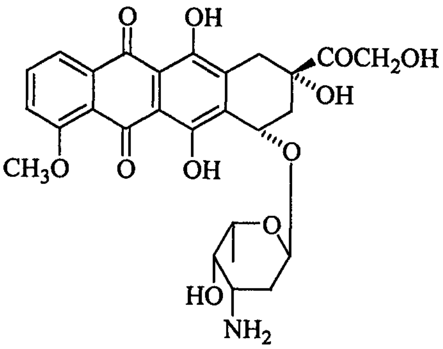

**17**

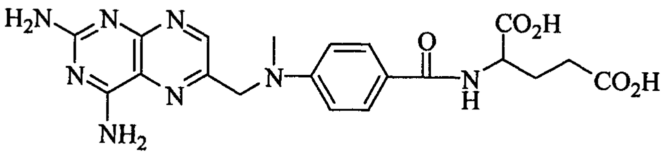

**18**

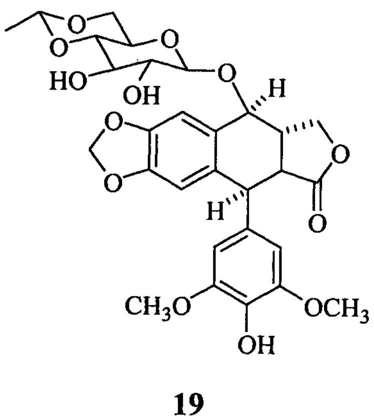

**19**

**Fig. 9:** Chemical structures of (**15**) 5-fluorouracil (5-FU), (**16**) taxol (TAX), (**17**) doxorubicin (DOX), (**18**) methotrexate (MTX), and (**19**) etoposide (ETO).

The cytotoxicity data for a series of isomeric triorganophosphinegold(I) n-mercaptobenzoates, $R_3PAu(n\text{-}SC_6H_4\text{-}CO_2H)$, are summarised in Table 4; cytotoxicity data for taxol are given in Table 3 for comparative purposes. From these data it is evident that while many of the gold compounds exhibited moderate to high levels of cytotoxicity, it was the 2-mercaptobenzoate series that proved to have the most interesting profiles. Particularly interesting was the suggestion that there might be some selectivity in the cytotoxic profile of the 2-mercaptobenzoates against the H226 (non-small cell lung cancer) and A498 (renal cancer) cancer cell lines. The $Et_3PAu(2\text{-}SC_6H_4CO_2H)$ compound was the most active of the gold compounds trialled and the order of cytotoxicity for $R_3PAu(2\text{-}SC_6H_4CO_2H)$ was R = Et > Cy > Ph. Particularly noteworthy was that in the case of H226 cell line, $Et_3PAu(2\text{-}SC_6H_4CO_2H)$ was more active than all of the established anti-tumour agents examined /36/. Having established these compounds possess cytotoxic activity, the challenge now is to conduct *in vivo* studies and these are underway at the time of writing.

### CONCLUSIONS

The use of the phosphinegold(I) thiolate, auranofin (**5**), in the treatment of rheumatoid arthritis indicates that this class of compound has potential for further development in medicinal chemistry. This is further borne out by the anti-tumour activity exhibited by **5**. Subsequent research, described above, has demonstrated that it is indeed possible to discover new anti-arthritic compounds with greater potency and reduced toxic side-effects, as judged by *in vivo* screening. Exciting developments also await discovery in the anti-tumour activity of phosphinegold(I) thiolates as cytotoxicity screening suggests that certain derivatives have very high potency and, perhaps even more importantly, there are indications of cancer cell specificity. Clearly, these preliminary results indicate the need for *in vivo* studies to confirm anti-tumour activity and acceptable toxicity profiles. Such developments as described above provide ample justification for further research activity in the field of metal-based drugs, in particular the investigation of gold compounds.

### ACKNOWLEDGEMENTS

The National University of Singapore (R-143-000-139-112) and the Biomedical Research Council Singapore (R-143-000-175-305) are thanked for support of on-going research conducted in Singapore. Previous work, conducted at The University of Adelaide, was supported variously by grants received from the Australia Research Council and The University of Adelaide.

### REFERENCES

1. Z. Guo and P. J. Sadler, *Angew. Chem. Int. Ed.*, **38**, 1512 (1999)
2. C. Orvig and M. J. Abrams, Editors of Special issue of *Chem. Rev.*, **99**(9), (1999)
3. W. F. Kean, F. Forestier, Y. Kassam, W. W. Buchanan and P. J. Rooney, *Sem. Arth. Rheum.*, **14**, 180 (1985)
4. A. J. Lewis and D. T. Walz, *Prog. Med. Chem.*, **19**, 1 (1982)
5. D. R. Haynes and M. W. Whitehouse, in K. D. Rainsford and G. P. Velo (Eds.), *New Developments in*

*Antirheumatic Therapy. Inflammation and Drug Therapy* Series. Volume III. Kluwer Academic Publishers, Dordrecht, 207 (1989)

6. E. R. T. Tiekink, *Crit. Rev. Oncol./Hematology*, **42**, 225 (2002)
7. M. Navarro, H. Pérez and R. A. Sánchez-Delgado, *J. Med. Chem.*, **40**, 1937 (1997)
8. M. Navarro, E. J. Cisneros-Fajardo, T. Lehmann, R. A. Sánchez-Delgado, R. Atencio, P. Silva, R. Lira and J. A. Urbina, *Inorg. Chem.*, **40**, 6879 (2001)
9. D. L. Shapiro and J. R. Masci, *J. Rheumatol.*, **23**, 1818 (1996)
10. M. Muranaka, T. Miyamoto, T. Shida, J. Kabe, S. Makino, H. Okumura, K. Takeda, S. Susuki and Y. Horiuchi, *Ann. Allergy*, **40**, 132 (1978)
11. C. F. Shaw III, *Chem. Rev.*, **99**, 2589 (1999)
12. P. Calabresi and R. E. Parks, in A. G.Gilman, L. S. Goodman, T. W. Rall and F. Murad (Eds.), *The Pharmacological Basis of Therapeutics*. MacMillan, New York, Sect. 13 (1985)
13. I. R. Garrett, M. W. Whitehouse, B. Vernon-Roberts and P. M. Brooks, *J. Rheumatol.*, **12**, 1079 (1985)
14. M. W. Whitehouse, A. H. Horewood and B. Vernon-Roberts, *Agents Actions*, Suppl., **24**, 184 (1988)
15. P. D. Cookson, E. R. T. Tiekink and M. W. Whitehouse, *Aust. J. Chem.*, **47**, 577 (1994)
16. M. W. Whitehouse, P. D. Cookson, G. Siasios and E. R. T. Tiekink, *Metal-Based Drugs*, **4**, 245 (1998)
17. C. S. W. Harker, E. R. T. Tiekink and M. W. Whitehouse, *Inorg. Chim. Acta*, **181**, 23 (1991)
18. J. F. Fries, D. Bloch, P. Spitz and D. M. Mitchell, *Am. J. Med.* **78** (Suppl. A), 56 (1988)
19. T. M. Simon, D. H. Kunishima, G. J. Vibert and A. Lorber, *Cancer*, **44**, 1965 (1979)
20. A. E. Finkelstein, O. R. Burrone, D. T. Walt and A. Misher, *J. Rheumatol.*, **4**, 245 (1977)
21. T. M.Simon, D. H. Kunishima, G. J. Vibert and A. Lorber, *J. Rheumatol.*, **6** (Suppl 5) 91 (1979)
22. T. M. Simon, D. H. Kunishima, G. J. Vibert and A. Lorber, *Can. Res.*, **41**, 94 (1981)
23. C. K. Mirabelli, R. K. Johnson, C. M. Sung, L. F. Faucette, K. Muirhead and S. T. Crooke, *Can. Res.*, **45**, 32 (1985)
24. C. K. Mirabelli, R. K. Johnson, D. T. Hill, L.F. Faucette, G. R. Girard, G. Y. Kuo, C. M. Sung and S. T. Crooke, *J. Med. Chem.* **29**, 218 (1986)
25. S. J. Berners-Price, G. R. Girard, D. T. Hill, B. M. Sutton, P. S. Jarrett, L. F. Faucette, R.K. Johnson, C. K. Mirabelli and P. J. Sadler, *J. Med. Chem.*, **33**, 1386 (1990)
26. G. D. Hoke, G. F. Rush, G. E. Bossard, J. V. McArdle, B. D. Jensen and C. K. Mirabelli, *J. Biol. Chem.*, **263**, 11203 (1988)
27. S. J. Berners-Price, R. J. Bowen, T. W. Hambley and P. C. Healy, J. Chem. Soc., *Dalton Trans*, 1337 (1999)
28. M. M. De Pancorbo, A. Garcia-Orad, M. Paz Arizti, J. M. Gutiérrez-Zorrilla and E. Colacio, *Metals Ions Biol. Med.*, **1**, 385 (1990).
29. M. P. Arizti, A. Garcia-Orad, F. Sommer F, L. Silvestro, P. Massiot, P. Chevallier, J. M. Gutiérrez-Zorrilla, E. Colacio, M. Martinez de Pancorbo and H. Tapiero, *Anticancer Res.*, **11**, 625 (1991)
30. A. Garcia-Orad, P. Arizti, F. Sommer, L. Silvestro, P. Massiot, P. Chevallier, J. M. Gutiérrez-Zorrilla, E. Colacio, M. Martinez de Pancorbo and H. Tapiero, *Biomed. Pharmacother.*, **47**, 363 (1993)
31. E. R. T. Tiekink, P. D. Cookson, B. M. Linahan and L. K. Webster, *Metal-Based Drugs*, **1**, 299 (1994)
32. L. K. Webster, S. Rainone, E. Horn and E. R. T. Tiekink, *Metal-Based Drugs*, **3**, 63 (1996)

33. D. Crump, G. Siasios and E. R. T. Tiekink, *Metal-Based Drugs*, **6**, 361 (1999)
34. E. R. T. Tiekink, *Metals Ions Biol. Med.*, **4**, 693 (1996)
35. B. R. Vincent, D. J. Clarke, D. R. Smyth, D. de Vos and E. R. T. Tiekink, *Metal-Based Drugs*, **8**, 79 (2001)
36. D. de Vos, P. Clements, S. M. Pyke, D. R. Smyth and E. R. T. Tiekink, *Metal-Based* Drugs, **6**, 31 (1999)
37. D. de Vos, D. R. Smyth and E. R. T. Tiekink, *Metal-Based Drugs*, **8**, 303 (2001)

